# Effects of a 60 Hz Magnetic Field Exposure Up to 3000 μT on Human Brain Activation as Measured by Functional Magnetic Resonance Imaging

**DOI:** 10.1371/journal.pone.0132024

**Published:** 2015-07-27

**Authors:** Alexandre Legros, Julien Modolo, Samantha Brown, John Roberston, Alex W. Thomas

**Affiliations:** 1 Human Threshold Research Group, Lawson Health Research Institute, London, Ontario, Canada; 2 Department of Medical Biophysics, Western University, London, Ontario, Canada; 3 Department of Medical Imaging, Western University, London, Ontario, Canada; 4 School of Kinesiology, Western University, London, Ontario, Canada; National Research Council, ITALY

## Abstract

Several aspects of the human nervous system and associated motor and cognitive processes have been reported to be modulated by extremely low-frequency (ELF, < 300 Hz) time-varying Magnetic Fields (MF). Due do their worldwide prevalence; power-line frequencies (60 Hz in North America) are of particular interest. Despite intense research efforts over the last few decades, the potential effects of 60 Hz MF still need to be elucidated, and the underlying mechanisms to be understood. In this study, we have used functional Magnetic Resonance Imaging (fMRI) to characterize potential changes in functional brain activation following human exposure to a 60 Hz MF through motor and cognitive tasks. First, pilot results acquired in a first set of subjects (N=9) were used to demonstrate the technical feasibility of using fMRI to detect subtle changes in functional brain activation with 60 Hz MF exposure at 1800 μT. Second, a full study involving a larger cohort of subjects tested brain activation during 1) a finger tapping task (N=20), and 2) a mental rotation task (N=21); before and after a one-hour, 60 Hz, 3000 μT MF exposure. The results indicate significant changes in task-induced functional brain activation as a consequence of MF exposure. However, no impact on task performance was found. These results illustrate the potential of using fMRI to identify MF-induced changes in functional brain activation, suggesting that a one-hour 60 Hz, 3000 μT MF exposure can modulate activity in specific brain regions after the end of the exposure period (i.e., residual effects). We discuss the possibility that MF exposure at 60 Hz, 3000 μT may be capable of modulating cortical excitability via a modulation of synaptic plasticity processes.

## Introduction

Human exposure levels to man-made time-varying magnetic fields (MF) have significantly increased since the industrial revolution. However, the mechanism and response of human physiology to extremely low frequency (ELF, < 300 Hz) MF exposure is still unclear, although research has shown that ELF MF exposure may modulate human physiology [1–6] and neurophysiology [7–11]. For instance, recent studies suggest that global head exposure to an ELF MF can modulate human electroencephalographic activity (EEG) and evoked potentials in healthy volunteers [7–9,11–13]. Furthermore, studies focusing on human motor control have demonstrated that ELF MF exposure can have an impact on motor behaviour in healthy volunteers, such as modifications in physiological tremor intensity and in spontaneous standing balance during exposure to 60 Hz and pulsed ELF MF respectively [14–17].

Organizations such as the International Commission on Non-Ionizing Radiation Protection (ICNIRP) and the Institute of Electrical and Electronics Engineers (IEEE) publish recommendations concerning maximum levels for safe exposure to ELF MF in order to protect both the general public and workers [18,19]. Previous basic restrictions from ICNIRP guidelines stated that the current density induced by MF during occupational exposure "… should be limited to fields that induce current densities less than 10 mA/m^2^" [20], corresponding to a computed MF value at the brain level of 1800 μT at a frequency of 60 Hz [21], the power-line frequency in North America. The 2010 ICNIRP guidelines no longer express the basic restrictions in terms of induced currents, but instead in terms of induced electric field [19]. With these new guidelines, the MF level required to reach the updated maximum basic restriction at the level of the head (estimated radius r = 0.1 m) for occupational exposure at 60 Hz (0.12 V.m^-1^) is now 6366 μT.

The previous results pointing towards modulations of postural oscillations [17,22,23] and physiological tremor [16,22,23], as a consequence of a time-varying MF exposure, suggest that the MF may have an effect on brain structures associated with these functions. The same assumption is made in studies reporting an impact of the exposure on cognitive performance [24,25]. Interestingly, the cerebral activation patterns associated with these cognitive and motor behaviours are well known and can be characterized using imaging technologies such as functional Magnetic Resonance Imaging (fMRI), using the so-called Blood Oxygen Level Dependant (BOLD) paradigm [26,27]. Functional MRI is indeed a non-invasive technique widely used to investigate brain function in general, allowing detection and quantification of brain activation patterns associated with specific cognitive or motor tasks [28–30]. It is therefore an interesting original imaging modality to investigate if, and how, ELF MF exposure may modulate brain activity patterns associated with both cognitive and motor functions.

In addition, correspondences between human EEG activity and fMRI activation are well established, and it is for example demonstrated that occipital EEG alpha activity (8–12 Hz band) is negatively correlated with functional activation measured using the BOLD fMRI paradigm [31]: BOLD activation is lower when alpha oscillations are stronger. Based on previous work reporting that exposure to ELF MF can increase EEG alpha oscillations of healthy volunteers [8,9], it is reasonable to extrapolate that it would result in a decreased functional activation if measured using a resting fMRI protocol (i.e when brain functional activity is measured at rest). However, fMRI is a technology that is best adapted to measure task-induced brain activation (i.e. motor or a cognitive task), which will be the focus of this paper.

For instance, in terms of motor tasks, the fMRI activation patterns associated with a finger-tapping task has been characterized in detail (see for example work by Sadato, Ibanez et al. in 1996 [29] and 1997 [28], as well as Toma and Nakai in 2002 [30]). The amplitude and frequency in a simple finger-tapping task are known to be positively correlated with the corresponding BOLD activation (Supplementary Motor Area (SMA), Primary Motor Area (SM1)) [27]. Also, EEG studies report that sensorimotor functions are associated with a decrease in alpha activity [32,33]. Interestingly, this also highlights the negative correlation between EEG and fMRI responses. Therefore, from the literature on the effects of ELF MF on human neurophysiology, suggesting that EEG alpha activity can be enhenced [9,34] and that physiological tremor and postural oscillations can be decreased by the exposure [16,17], we hypothesized that a 60 Hz MF exposure should decrease the spontaneous tapping frequency in an index-to-thumb finger tapping task performed at natural frequency, and should be associated with a decrease in the corresponding BOLD activation using fMRI.

In terms of cognitive tasks, the mental rotation task is a well-characterized, standard test consisting in discriminating if two images representing 3D geometrical shapes are identical but rotated in space or different [35]. Functional MRI studies have identified the activation of a network of brain regions involved in this task, which include the cerebellum, the premotor cortex and the superior parietal lobule [36–38]. Interestingly, one study investigating the effect of a 600 μT, 50 Hz MF in humans found out a decreased performance in attentional and memory tasks [39]. More recently, Corbacio et al. [25] reported that the improvement associated with the repetition of a short term memory task no longer existed after a one-hour exposure to a 3 mT, 60 Hz MF. Based on these reports of impaired performance associated with MF exposure, we hypothesize that a 60 Hz MF exposure will result in a decreased functional activation of the brain regions involved in the mental rotation task, along with a decrease in the associated performance.

This paper first presents the results from a pilot study aiming to demonstrate the technical feasibility and validate the use of fMRI as an appropriate imaging tool in a bioelectromagnetics study. This pilot protocol tested the effect of a 30-minute, 60 Hz MF exposure at 1800 μT (the maximum MF flux density value in 1998 ICNIRP basic restrictions [20]). Second, it reports results, for a larger cohort, involving the execution of two tasks (motor, finger tapping; and cognitive, mental rotations) before and after exposure to a one-hour 3000 μT MF at 60 Hz. We discuss the implications of the observed functional brain activity modulation in terms of biological effects and the mechanisms involved.

## Methods

### Participants

Twenty-nine healthy right-handed volunteers (N = 29, mean age = 26.7±1.36) were tested in a pseudo double-blind experiment (i.e. the experimenter discovered the exposure condition, “control” or “exposed”, only after the end of direct interaction with the participant). This study was approved by the Health Sciences Research Ethics Board (HSREB) of Western University (ethics approval #13460E). After participant recruitment (posted advertisements for student volunteers at Western University, London, Ontario, Canada), participants gave their written informed consent. Exclusion criteria for participants included a self-reported history of serious medical illness, drug or alcohol abuse, head or eye injury involving metal fragments or any magnetic/electrical implants. All participants abstained from caffeinated beverages, alcohol, or nicotine consumption at least 24 hours before the time of the experiment. Prior to the experiment, a standard MRI screening questionnaire was given to participants to ensure their safety upon entry to the MRI unit. Participants were also asked to complete the Oldfield handedness questionnaire [40] to indicate their handedness.

Of the 29 healthy participants who were recruited and participated in the study, 20 datasets from these participants (average age = 25.87 ± 5.9) were usable for the finger-tapping portion of the experiment. Of these 20 participants, 11 were “control” and 9 were “exposed” to the 3000 μT, 60 Hz MF. For the mental rotation portion of the experiment, 21 datasets were usable (average age = 25.3 ± 5.6). Of these 21 participants, 11 were in the “control” group (6 males, 5 females) and 10 in the “exposed” group (5 males, 5 females). Dataset exclusions were due to the following: excessive movement during imaging (n = 4), technical issue preventing finger tapping recording (n = 1), inability to see pictures without glasses during the mental rotation task (n = 2), alcohol during the 24 hours preceding the experiment (n = 1), and a drop-out (n = 1).

### Apparatus

The experiment was run using a 3.0 Tesla Magnetic Resonance Imaging (MRI) Scanner (Siemens Verio, Erlangen Germany) and a 32-channel head coil. The MF exposure was produced by the MRI Z-gradient coil (programmed by our medical physicist, Dr. Jean Théberge, and Siemens Medical Ltd, Canada). An MRI-compatible button press system taped to a glove on the participant’s right was used to record participant response to visual stimuli (go/no-go). Visual stimuli for both tasks were displayed on an MRI-compatible projection screen using a custom-made LabView (National Instruments, Austin, USA) program. Participants were instructed when to tap by displaying "TAP" or "STOP" on the screen seen by the participant from inside the MRI using a mirror attached to the head coil. The LabView program also recorded tapping times and mental rotation answers.

### Functional Magnetic Resonance Imaging (fMRI)

Thirty non-overlapping, oblique slices (5 mm thickness, 5% gap) that covered the cerebrum and cerebellum were imaged using a gradient echo planar imaging (EPI) sequence (parameters: TE = 50 ms; TR = 3000 ms with 500 ms delay; matrix 64 x 64; flip angle = 90°). 110 whole brain scans were collected with a 192 mm field of view and 3x3x5 mm voxels (parameters constant between functional runs). High-resolution T1-weighted anatomical images (1 mm isovoxel) were also collected with an MP-RAGE (magnetization prepared radio-frequency pulses and rapid gradient-echo sequence, with the following parameters: TR = 1800 ms, matrix size = 256 x 256, slices ranged between 160 and 192) to co-register these anatomical images with functional images.

### Experimental procedure

**Fig 1:**
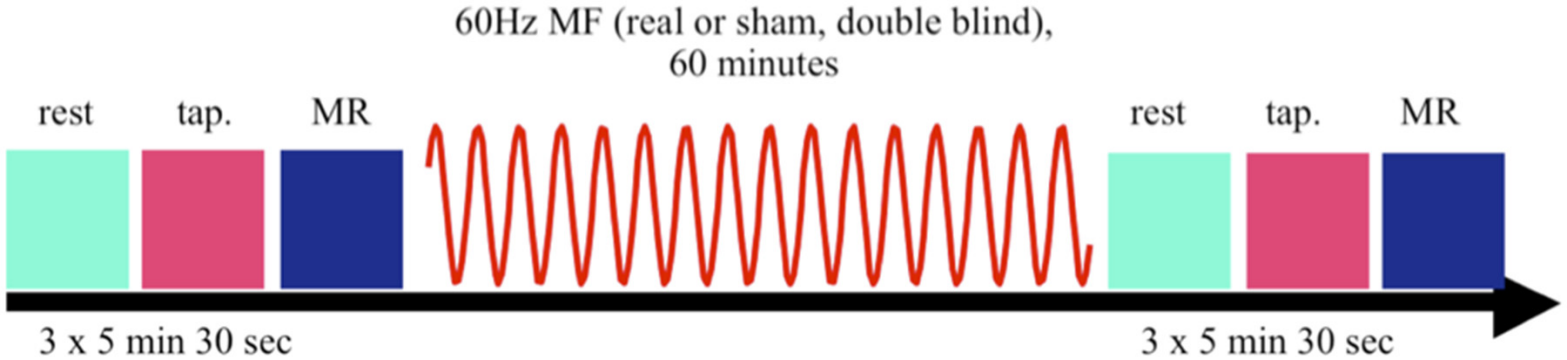
Time course of experiment. Sequence of imaging and testing periods, including one hour of control or 60 Hz MF exposure to a 60 Hz MF at 3000 μT during one hour.

The two-hour MRI session included three conditions: 1) rest, 2) finger tapping, and 3) mental rotation; which were performed Pre- and Post- exposure as shown in Fig 1. Participants were placed in the MRI scanner in a head-first supine position with their heads gently restrained in a 32-channel phased array head coil lined with foam padding to ensure minimal movement during the scan. The experiment began with the acquisition of an anatomical image. Participants then completed the three Pre-exposure conditions (rest, finger-tapping and mental rotation). First, during the rest period, subjects were asked to close their eyes and let their mind wander. Second, during the finger-tapping task, subjects were asked to rhythmically tap the index finger to the thumb of the dominant hand at a natural and comfortable frequency. This choice was made to avoid spurious brain activation induced by any possible cue guiding the finger tapping frequency, and it offered the possibility to test the potential impact of the exposure on the spontaneous tapping frequency, which would be associated with a change in the corresponding BOLD activation [27,30]. This task is routinely used in fMRI studies due to the robustness of induced brain activation [41]. Since the production of functional images requires comparing periods of activity to periods of rest, the task was performed in alternating 15-second blocks. Third, participants were presented with two 3-dimensional objects represented in 2D (i.e. an image of a 3D shape), projected on a screen side-by-side (mental rotation task). Participants were asked to mentally compare the objects and determine as quickly as possible, if: a) the objects are identical (rotated by 0°, 30°, 60°, or 90°), or b) the objects are mirror images (rotated by 0°, 30°, 60°, or 90°). The goal of the task is to determine both the rate of spatial processing and intelligence [42,43]. Specifically, subjects were rated on the speed of responses and how accurately they could distinguish between mirrored and non-mirrored images. Using fMRI, brain regions consistently associated with the mental rotation task have been identified as: posterior parietal cortex (intraparietal sulcus, Broadmann’s area 7), middle frontal gyrus, extra-striate cortex, hand somatosensory cortex, and frontal cortex [37,44,45]. The order of the two tasks (finger tapping and mental rotation) was kept identical for each subject. This design choice was made to avoid an additional source of experimental variability (i.e. the order of presentation effect), in order to maximize the probability to find an effect, if it exists. Since our main focus was to extend our pilot results, the finger tapping task was always performed first after the one hour exposure period; acknowledging the possibility of decreasing the probability to identify an effect in the mental rotation task if it exists.

Functional data was then acquired for each of the three conditions. Then, the one-hour 60 Hz MF sequence was delivered (exposure or control). After the 60-minute exposure period, participants repeated the same three tasks under the same three imaging sequences. Upon removal from the MRI, participants completed a Field Status Questionnaire (FSQ) [46]. This questionnaire evaluated whether participants were able to detect the presence of the 60 Hz MF, as well as the degree of certainty in doing so, and the level of comfort and stress felt during the study. The time course of the experiment is summarized in Fig 1.

### 60 Hz magnetic field exposure

**Fig 2:**
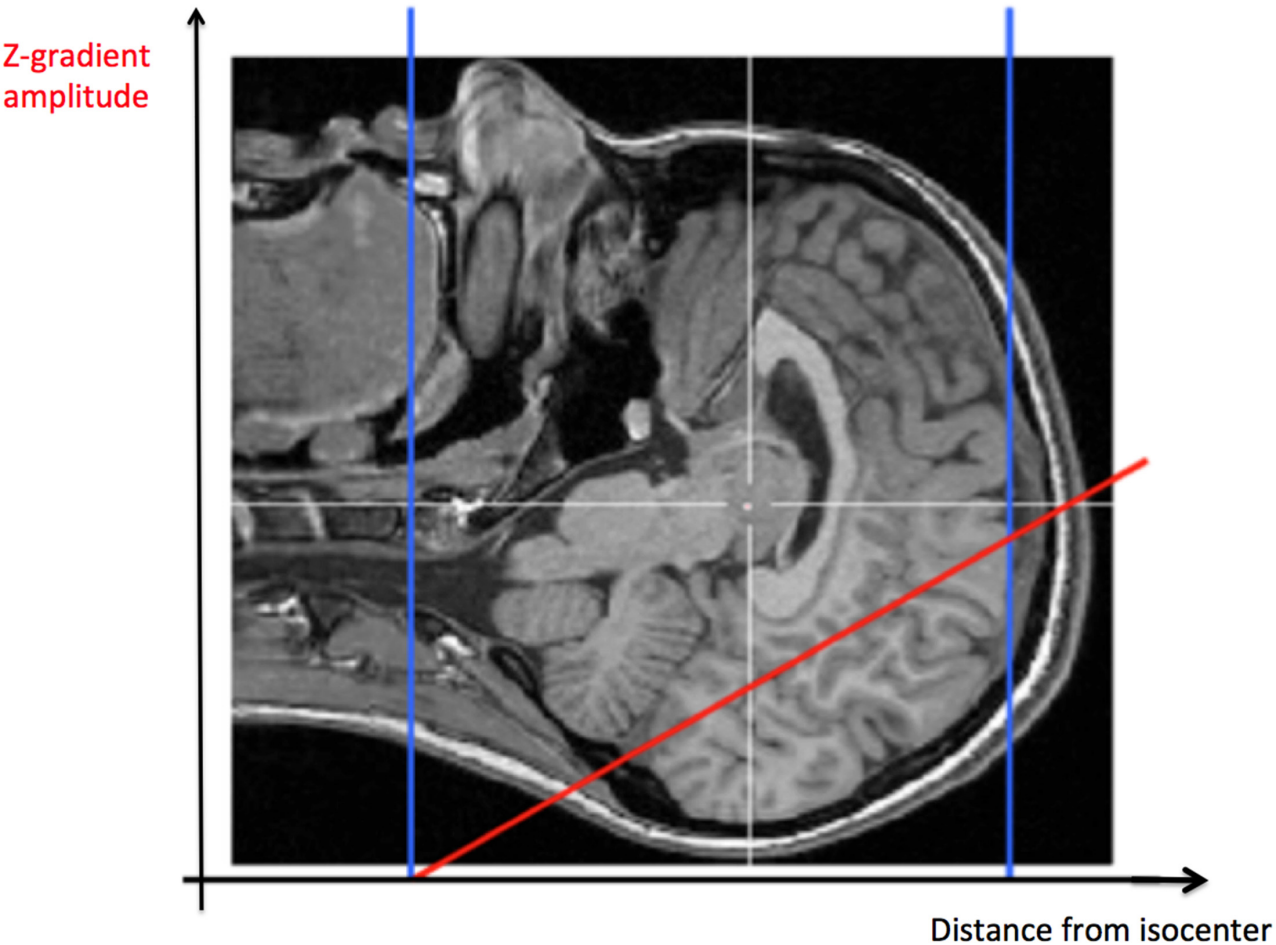
Magnetic field gradient. The maximal MF level was obtained at the cortical level. The variation of the MF intensity depends on the position along the bore Z-axis and is shown in red. The MF intensity delivered by the gradient coil linearly decreases to reach zero at the isocentre (at the level of the first cervical vertebrae).

In the active 60 Hz MF condition, participants were exposed to a 60-minute MF at 60 Hz and 3000 μT within the bore of the MRI unit. The 60 Hz MF exposure was generated by the Z gradient coil of the MRI scanner, and the highest time-varying magnetic field flux density was at the top of the cortex (magnetic field flux density of 3000 μT at 1 cm underneath the skull, see Fig 2). Since the gradient coil of the MRI normally generates ‘zero gradient field’ at the isocentre of the MR bore during normal operation and imaging (see [47]), the patient table was moved horizontally to expose the entire brain to the 60 Hz MF generated by the Z gradient coil. Note that this MF exposure procedure using the Z gradient coil has been used on the same MRI scanner in a previous study testing the impact of a specific pulsed 200 μT MF on human volunteers [48]. It is important to remind here that, when the gradient coil is not active, the MF present at the isocentre is still 3 T (static—as the static MF from an MRI is always present). However, the gradient coils are only activated during imaging are exposure sequences, and are not active the rest of the time. Also, the principle of using the Z gradient coil to deliver the exposure was used in a previous study from our group [48]. In the control condition, participants were moved to the same position as the exposed group and an audio clip mimicking the sound of the 60 Hz MF was played in the MR room to ensure similar experimental conditions during the 60-minute MF exposure period.

The MF exposure considered in the present study was qualitatively similar to the MF generated by a power-line in a real-life situation (sinusoidal at the same frequency– 60 Hz, spatial decay of the MF with distance), and at least partly quantitatively similar (same order of magnitude for the MF to which electricity workers can be exposed to). However, the spatial decay was quantitatively different (constrained by the technical specifications of our MRI system), and obviously the strong static MF at 3T that was generated by our scanner would not be present in a real-life situation. Furthermore, since both groups are exposed to the exact same MRI static field and imaging sequences, any potential difference observed in functional brain activation will be due to the 3 mT, 60 Hz MF exposure. This difference might result either from the 60 Hz MF exposure only, or from the addition of the 60 Hz, 3 mT MF to a 3T background static field.

### Data analysis

Regions of interest (ROI) were chosen using a priori information regarding the brain regions associated with the tapping task [28–30]. fMRI data files were analyzed using the software BrainVoyager (BrainVoyager QX 1.9.10, Brain Innovation, The Netherlands). First, functional data were pre-processed to compensate for head movement and signal drift, using a built-in 3D motion correction algorithm and temporal filtering. The quality of head movement correction was systematically inspected after this processing step for each set of fMRI images, using the movement curves and video files provided by BrainVoyager QX. Second, spatial smoothing was applied using a built-in 3D Gaussian smoothing of 8 mm Full Width at Half Maximum to improve the reliability of comparisons between participants. Functional images were then co-registered with the anatomical T1 weighted images. Third, co-registered images were normalized into the Talairach space. Finally, functional images were evaluated with BrainVoyager using a general linear model (GLM) multi-study analysis with a value of p < 0.001 (Bonferroni-corrected value). Please note that functional activation maps were calculated in the Talairach space, while anatomical images on which they are co-registered for illustration purposes were not. Therefore, since the anatomical image used here corresponds to an individual participant and because there is inter-participant variability in brain anatomy, there may be visual inconsistencies between the activation regions determined according to their Talairach coordinates and the corresponding location on the illustrations.

Pre-exposure activation maps were produced for all participants to determine if our results replicated previously published BOLD fMRI studies of the finger-tapping task [38,44] and the mental rotation task [37,44,45]. ‘Post- minus pre-exposure’ images were produced for each of the control and 60 Hz MF conditions for each task. Beta weights, evaluating the intensity of the correlation between a predictor function (on/off) relative to the task and the measured BOLD activity in given brain voxels, were then extracted from each ROI. Beta weights for each ROI were analyzed separately using SPSS (SPSS 16.0, SPSS Inc., Chicago, USA), using a mixed design ANOVA for repeated measures with a between-subjects factor (exposure group). For this study, p < 0.05 was considered significant and was reported as a ‘non-significant trend’ when 0.05 ≤ p ≤ 0.1. Bonferroni corrections accounting for multiple comparisons were systematically integrated in the statistical analyses conducted with SPSS.

Button press data from the tapping task were analyzed using Matlab (The Mathworks, USA) to determine the frequency (inverse of the time period between two button presses) and regularity of the recorded responses. Similarly, for the mental rotation task, the task performance (percentage of correct answers) was calculated from the button press data using Matlab. A repeated measures ANOVA (within subjects: time; between subjects: condition) was applied to determine if there were significant differences in tapping frequency between groups before and after the 60 minute exposure period for both groups (“control” and “60 Hz MF”) using SPSS. Finally, a χ-squared test was applied to the FSQ results for both groups to evaluate the potential capability of participants to detect the presence of the 60 Hz MF.

### 60 Hz 1800 μT MF Pilot experiment—specific methods

In this section, we mention the differences in methodology in our pilot experiment (N = 9 at 1800 μT) compared to the full study (N = 20 and N = 21 respectively for the finger tapping and mental rotation tasks at 60 Hz, 3000 μT). For this pilot experiment, nine healthy right-handed volunteers participated in this experiment (mean age = 25.5; range = 21–33; 5 women) after giving written informed consent according to the guidelines of the Western University Health Sciences Research Ethics Board (#11956E). Participants were randomly assigned to one of two conditions (5 60 Hz MF and 5 control—one subject dropped out) after the subject was installed in the scanner.

This pilot experiment was run on a 1.5 T whole-body MRI scanner (Siemens Avanto, Erlangen, Germany) with a 12-channel phased array head coil. Once in the MRI, each participant completed a 1-hour and 10 minute testing session including (1) a set of head localizing images, (2) 5 minutes and 30 seconds of the rhythmic index-to-thumb opposition task with functional imaging (BOLD), (3) 30 minutes of rest during which the participant may or may not have been exposed to the 60 Hz MF, (4) a second period of 5 minutes and 30 seconds of rhythmic index-to-thumb opposition task with functional imaging, and (5) 10 minutes of anatomical image collection. Participants were instructed when to start and stop tapping via the intercom using the words ‘Start’ and ‘Stop’. The 60 Hz 1800 μT MF was produced using the same custom software MRI gradient system method as the 60 Hz 3000 μT MF in the full experiment (using the Z gradient coil, please contact the corresponding author to place a request for this Siemens Gradient System procedure for research purposes) and as previously validated [48].

ROIs selected were: contralateral (left) primary somatosensory cortex (S1; Talairach coordinates: x = -37; y = -29; z = 58), the contralateral (left) anterior cingulate cortex (AC, in its posterior section; Talairach coordinates: x = -8; y = -3; z = 41), and the ipsilateral (right) cerebellum (Talairach coordinates: x = 14; y = -52; z = -18).

## Results

### Pilot results—1-hour exposure at 1800 μT

FSQ results showed that subjects in both groups were unable to judge whether or not they were being exposed or control exposed to the MF (χ2 = 2.8, df = 1, p > 0.05) and none of the subjects in either group had a high confidence level in their judgements (maximum 2 out of 5). A χ2 test is a statistical non-parametric test aiming to provide the significance of the difference between expected and observed distributions.

**Fig 3:**
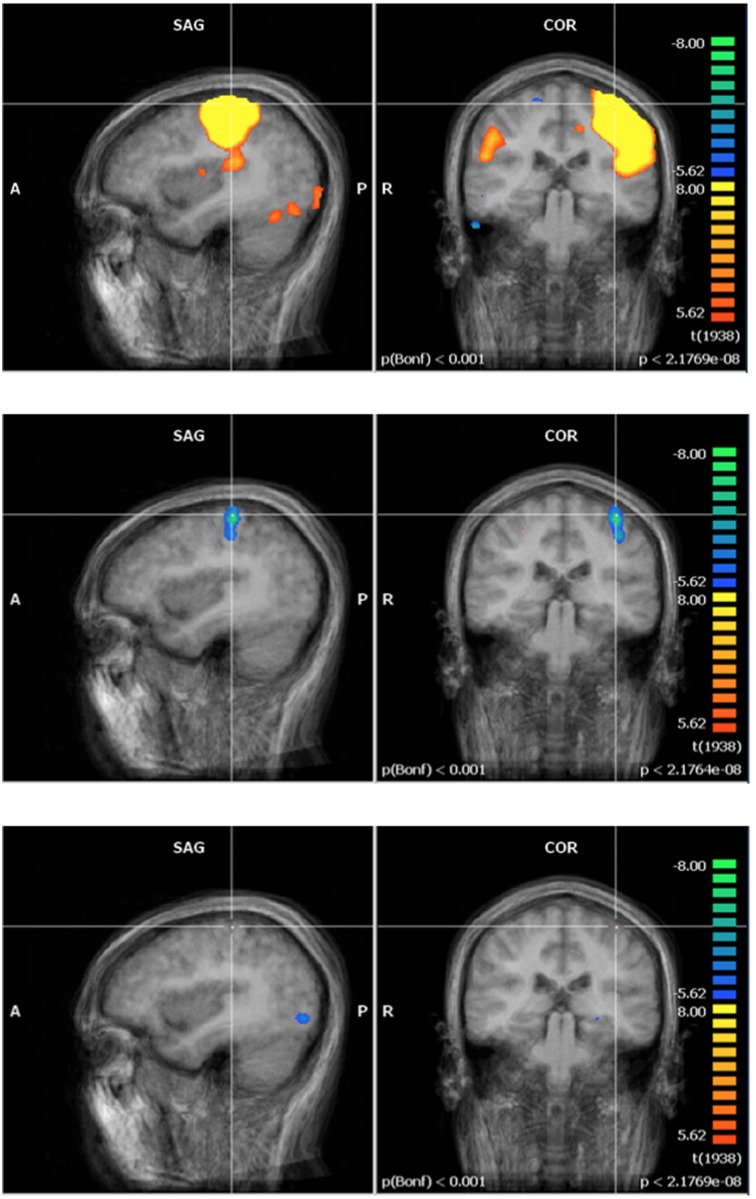
Functional brain activation—primary somatosensory cortex (S1). S1: Top row—Tapping: pre-exposure group image (N = 9). Middle row—Tapping: post- minus pre-exposure condition (control, N = 5). Bottom row—Tapping: post- minus pre-exposure condition (60 Hz MF, N = 4). Results centered on the point of Talairach coordinates (X = -40, Y = -31, Z = 52).

**Fig 4:**
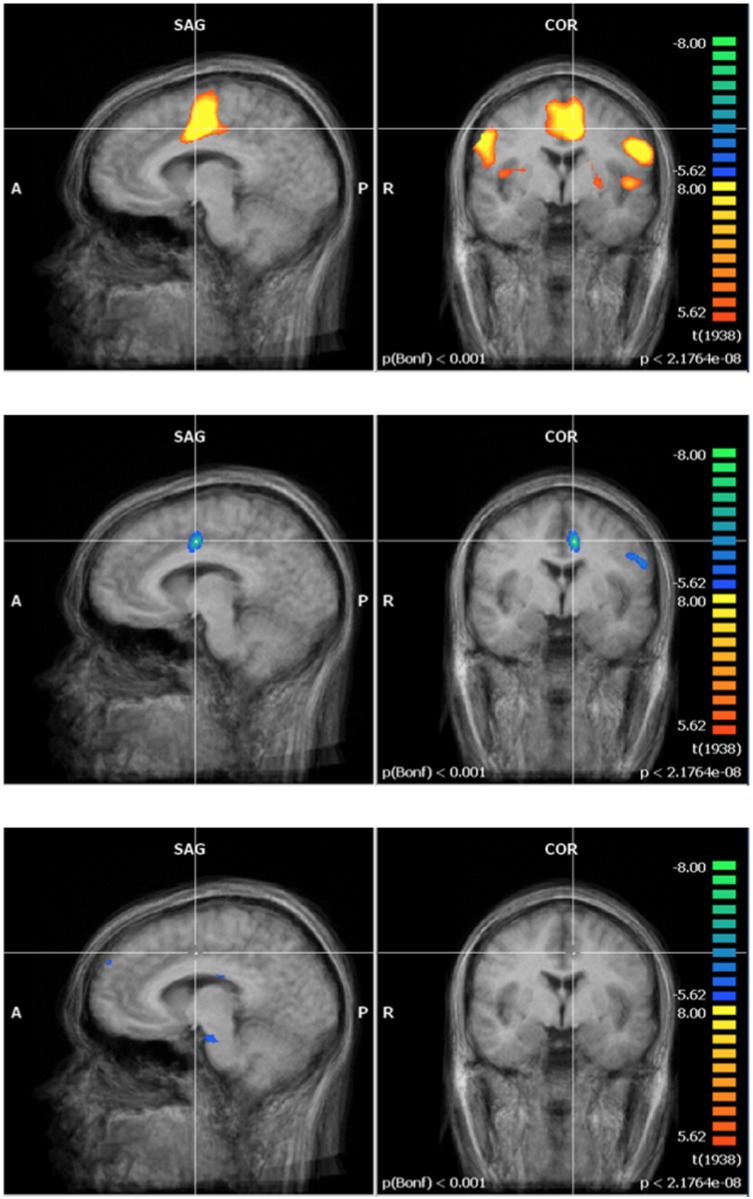
Functional brain activation—anterior cingulate cortex. AC: Top row—Tapping: pre-exposure group image (N = 9). Middle row—Tapping: post- minus pre-exposure condition (control, N = 5). Bottom row—Tapping: post- minus pre-exposure condition (60 Hz MF, N = 4). Results centered on the point of Talairach coordinates (X = -7, Y = -6, Z = 39).

**Fig 5:**
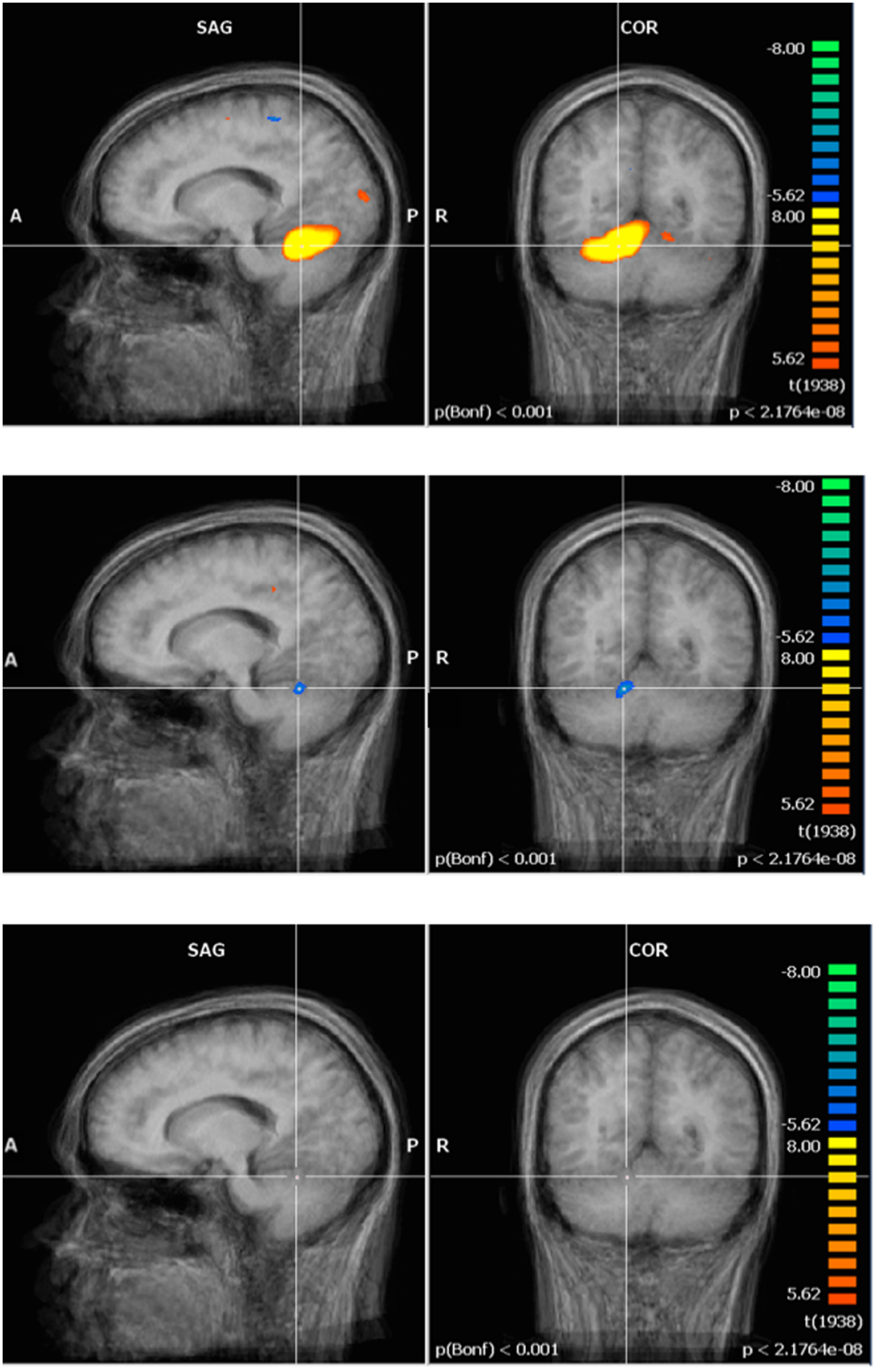
Functional brain activation—cerebellum. Cerebellum: Top row—Tapping: pre- exposure group image (N = 9). Middle row—Tapping: post- minus pre-exposure condition (control, N = 5). Bottom row—Tapping: post- minus pre-exposure condition (60 Hz MF, N = 4).

Regarding fMRI data, the pre-exposure group image revealed activation in three regions covering several brain structures each (Figs 3–5, top row): first, the contralateral primary and supplementary motor cortex (M1 and SMA) and the contralateral S1; second, bilaterally, the medial section of the premotor cortex, and the posterior section of the AC; third, the anterior lobe of the ipsilateral cerebellum. Post- minus Pre-exposure comparison images were produced for each experimental group (GLM analysis from Brain Voyager—Figs 3–5, middle row for the control exposure group, bottom row for the 60 Hz MF exposure group) and showed deactivation in the contralateral S1 and AC, and anterior lobe of the ipsilateral cerebellum in the control group (Figs 3–5, middle row). Surprisingly, no difference of activation Post-exposure as compared to Pre-exposure was found for the exposed group (Figs 3–5, bottom row). A within-subjects ANOVA with a between-subjects factor (group) conducted on beta weight values extracted from these ROI showed a significant decrease in activation for Post- as compared to Pre-exposure in the S1 (F = 6.8, p < 0.05, main effect), and the cerebellum (F = 6.8, p < 0.05, main effect), while the activation decrease in the AC approached significance (F = 4.85, p = 0.063). A significant time by exposure interaction was seen in the AC, confirming that post-exposure deactivation was stronger in the control group than in the exposed group (F = 12.04, p < 0.05). The time by exposure interaction was not significant either in the cerebellum (F = 1.548, p > 0.2) or in S1 (F = 1.548, p > 0.2).

### Motor task results—Finger tapping before and after 1 hour exposure at 3000 μT

Similar to our pilot results, participants were unable to detect the 60 Hz, 3000 μT MF as shown with the results of the χ-squared test (FSQ: χ^2^ = 3.23, p > 0.05; level of certitude = 2 out of 5). In addition, one may note that the participants were not confident in their judgment (average level of confidence: 2 out of 5). The repeated measure analysis on the button press data revealed no significant changes in the variability of the mean period between two button presses. Indeed, a decrease in tapping variability occurs over the time course of the experiment, for both groups, with a non-significant difference in amplitude (not shown). The repeated measure analysis on the button press data revealed no significant changes in the mean period either.

**Fig 6:**
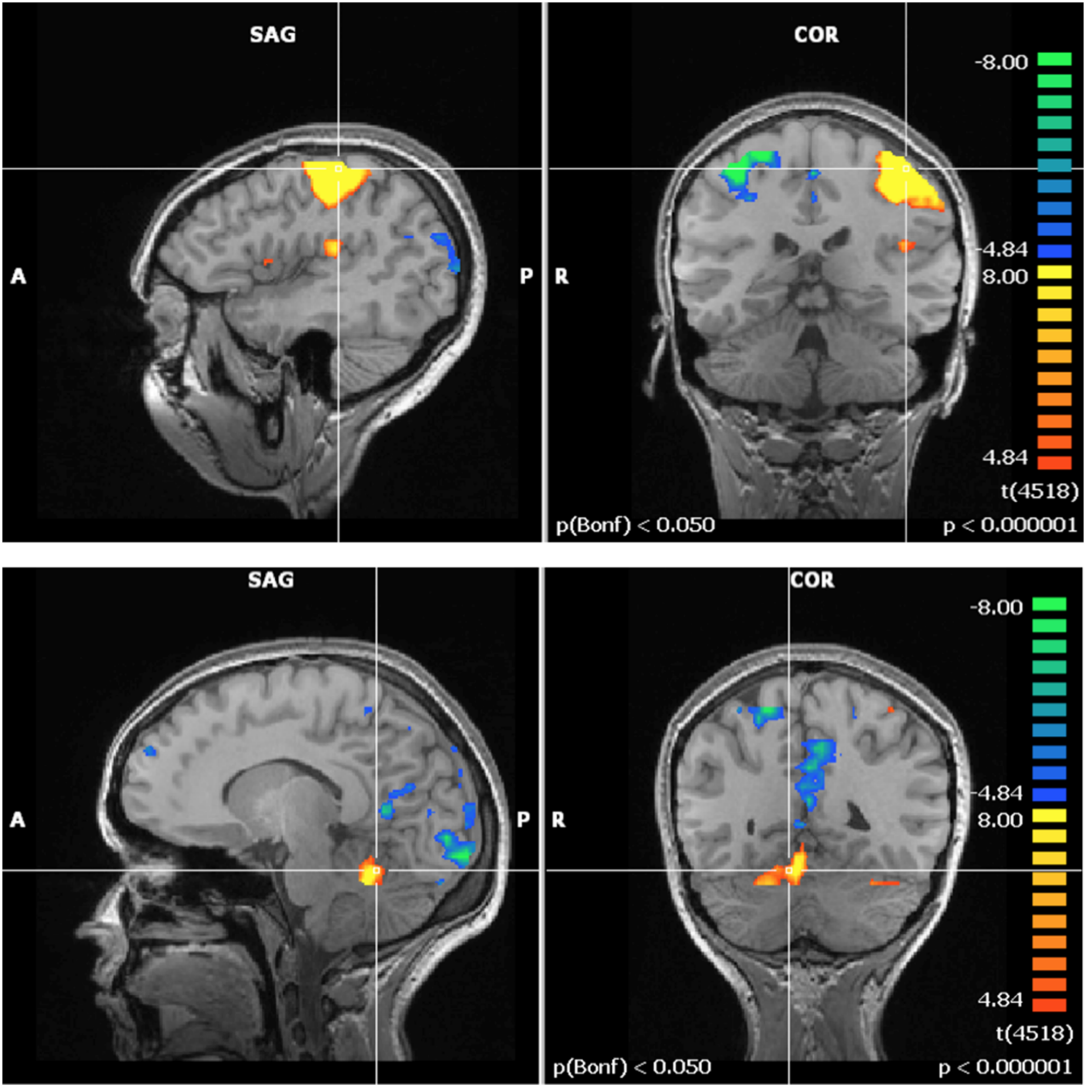
Pre-exposure finger tapping averaged activation map of the contralateral motor cortex regions (top row) and the ipsilateral cerebellum (bottom row) for 20 participants for the full study at 3000 μT. The contralateral motor cortex images (top row) presented are centered on the Talairach coordinates corresponding to S1 (x = -41, y = -31, z = 53) for the motor cortex region. The ipsilateral cerebellum images (bottom row) presented are centered on the Talairach coordinates corresponding to the anterior lobe of the ipsilateral cerebellum (x = 18, 7 = -47, z = -15).

Functional activation maps generated by Brain Voyager using the procedure described above (data analysis section) revealed that the finger-tapping task was inducing robust activation of the contralateral premotor cortex (PM), M1, SMA, S1 and the anterior lobe of the ipsilateral cerebellum. Activation of M1 and of the anterior lobe of the ipsilateral cerebellum by the finger-tapping task is illustrated in Fig 6. Note that the images presented in this section are centered on Talairach coordinates corresponding to S1 (x = -41, y = -31, z = 53) for the motor cortex region and on the anterior lobe of the ipsilateral cerebellum (x = 18, y = -47, z = -15).

**Fig 7:**
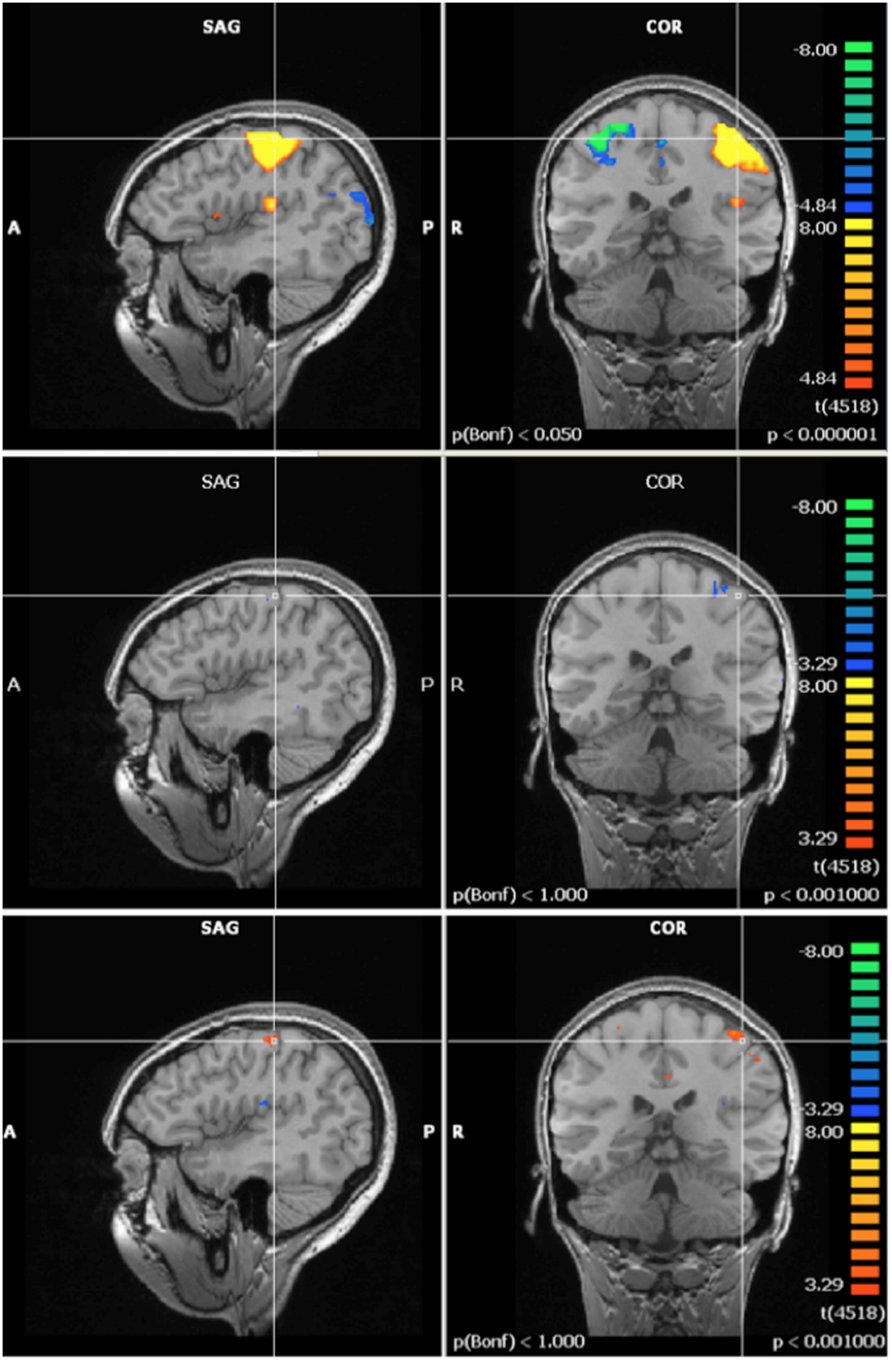
Increased activation in S1 in the 60 Hz MF exposure group. *(All images were normalized in Talairach space—BrainVoyager GLM analysis). Results centered on the point of Talairach coordinates (X = -40, Y = -31, Z = 52). Fig 7. Top) Pre-exposure activation and deactivation for all subjects (N = 20). Fig 7. Middle) Post—minus- pre control exposure (N = 11). Fig 7. Bottom) Post- minus- pre 60 Hz MF exposure (N = 9).

**Fig 8:**
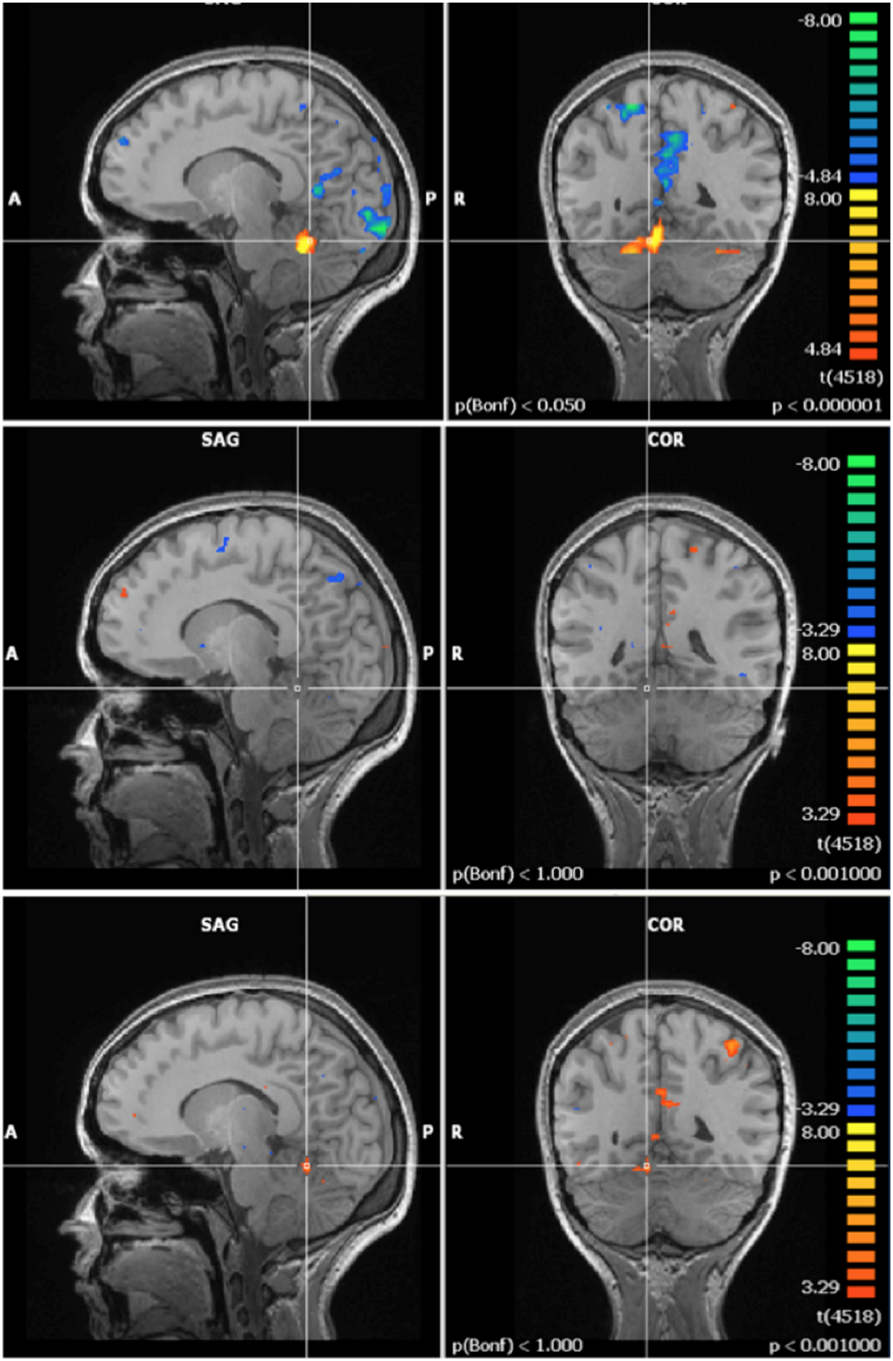
Increased activation in the anterior lobe of the ipsilateral cerebellum in the 60 Hz MF exposure group (All images were normalized in Talairach space—BrainVoyager GLM analysis). Results centered on the point of Talairach coordinates (X = 18, Y = -47, Z = -15). Fig 8. Top) Pre-exposure activation and deactivation for all subjects (N = 20). Fig 8. Middle) Post-minus-pre control exposure (N = 11). Fig 8. Bottom) Post-minus-pre 60 Hz MF exposure (N = 9).

In order to detect potential modulation in activation patterns by the 60 Hz MF exposure, Post-exposure (i.e. exposed or control) activation maps were subtracted from Pre-exposure maps in each experimental group. Interestingly, Post- minus Pre-exposure activation maps revealed differences between the control and exposed groups. In the control group, no significant difference (GLM analysis) in brain activation was observed before as compared to after the 1-hour resting period (see middle row of Figs 7 and 8 for the motor cortex region and the cerebellum, respectively). Conversely, in the exposed condition, the GLM analysis (at p < 0.001) highlighted significant differences in functional activation between Pre- and Post-exposure in S1 (F = 3.872; p = 0.00023) and in the anterior lobe of the ipsilateral cerebellum (F = 3.722; p = 0.0003), as illustrated in the activation maps on the bottom row of Figs 7 and 8.

Overall, these results show that functional brain activation induced by finger tapping, measured using the BOLD paradigm, is significantly higher in the contralateral S1 and in the ipsilateral cerebellum (anterior lobe) after MF exposure, as compared to after control exposure. This is consistent with our pilot results in which exposed participants had a significantly higher functional activation Post- than Pre-exposure in the exposed group as compared to the control group.

### Cognitive task results—Mental rotation task before and after 1-hour exposure at 3000 μT

FSQ results indicate that no participants in either group could determine whether they had been exposed to the 60 Hz MF or control exposure (FSQ: χ2 = 1.718, p > 0.05; average level of certainty = 2.8 out of 5), in line with pilot results. Similarly to the finger-tapping task, analysis of the button press data did not reveal any significant differences in the speed or accuracy of responses between Pre- and Post-exposure in both control and 60 Hz MF exposure groups.

**Fig 9:**
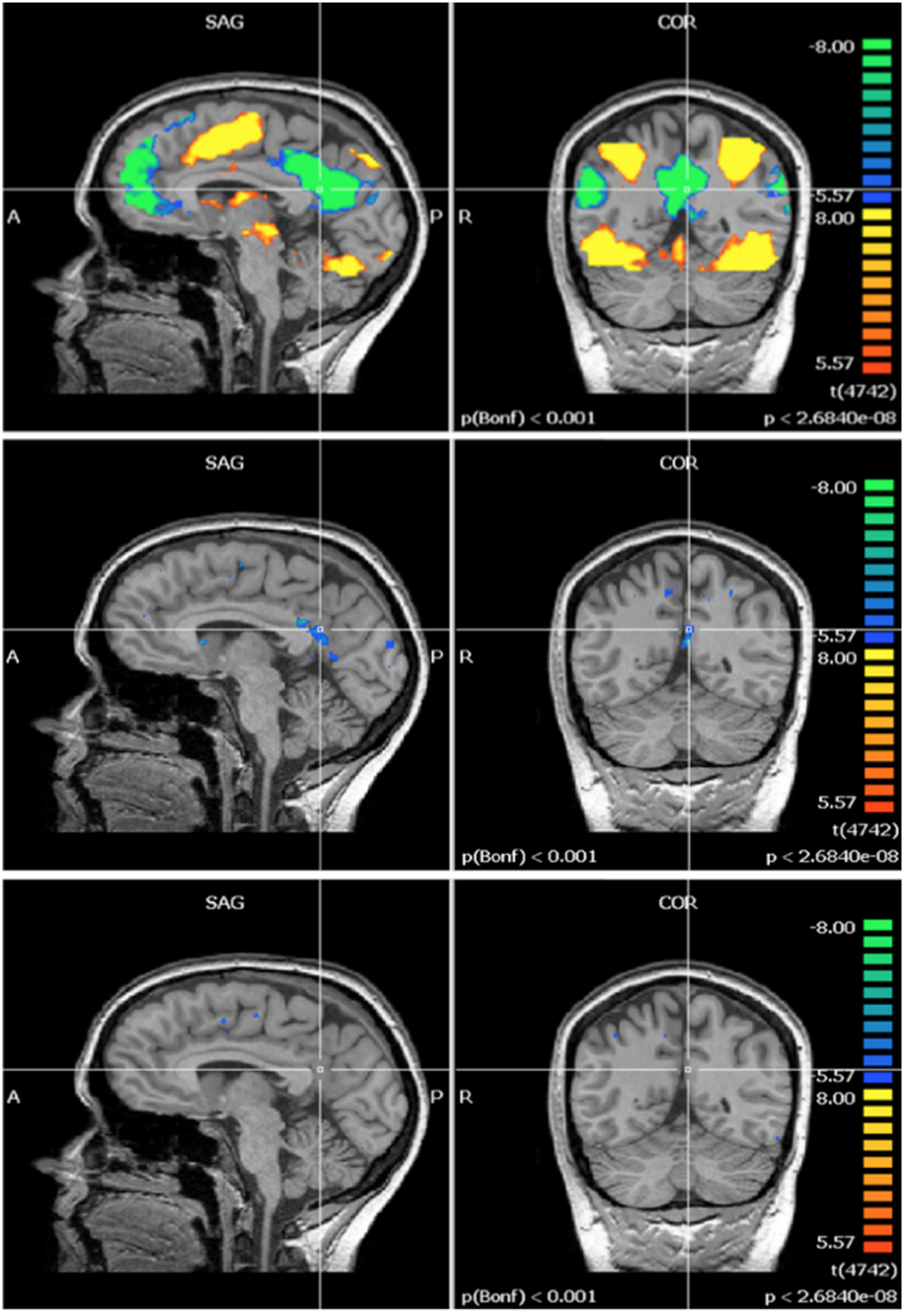
Activation in the posterior cingulate during the mental rotation task. Top) Pre-exposure (N = 21); Middle) Post- minus pre- exposure in the control group (N = 11); Bottom) Post- minus pre- exposure in the 60 Hz MF exposure group (N = 10). Results centered on the point of Talairach coordinates (X = -5, Y = -53, Z = 16).

**Fig 10:**
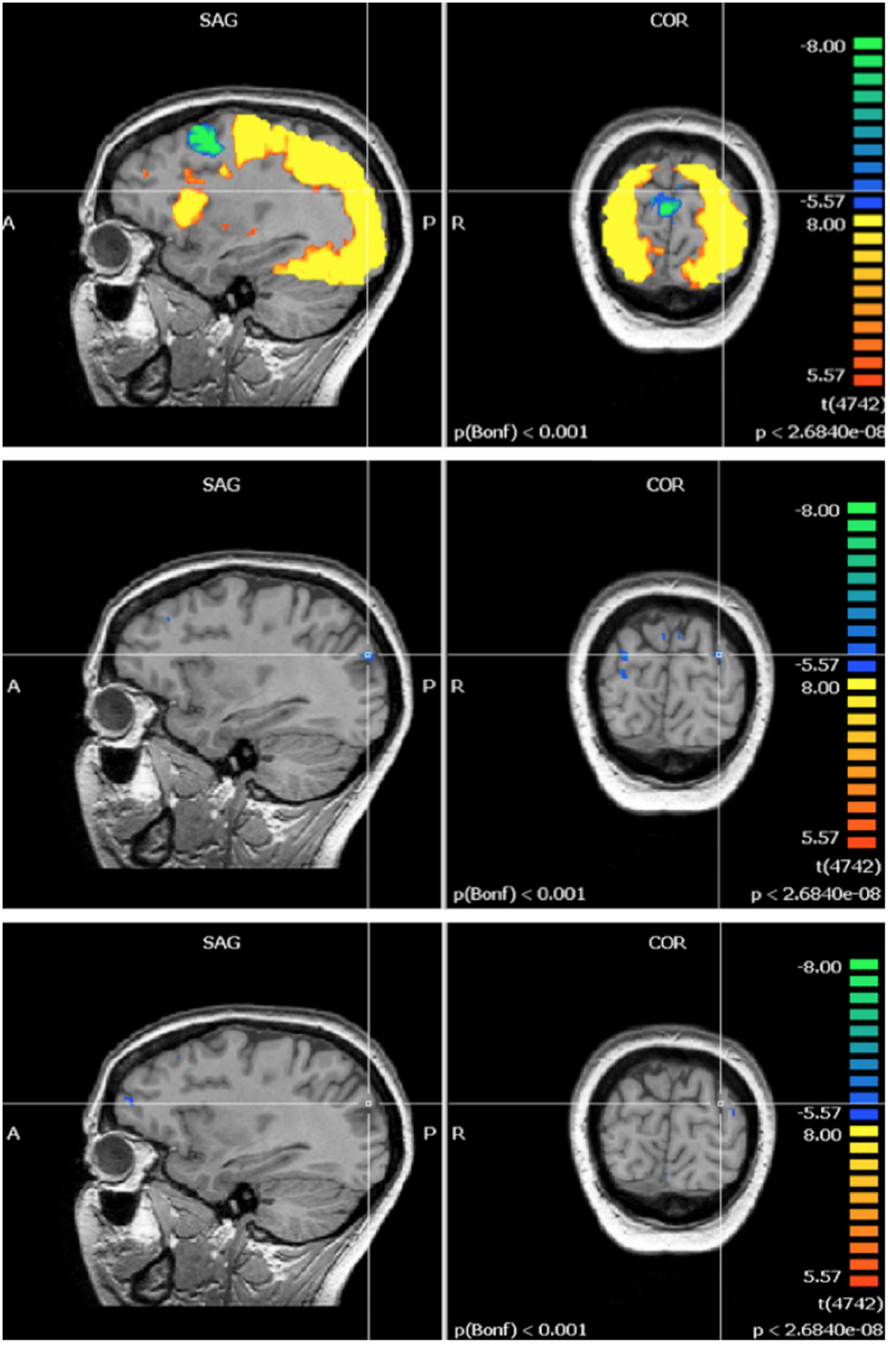
Activation in the left intraparietal sulcus during the mental rotation task. Top) Pre-exposure (N = 21); Middle) Post- minus pre- exposure in the control group (N = 11); Bottom) Post- minus pre- exposure in the 60 Hz MF exposure group (N = 10). Results centered on the point of Talairach coordinates (X = -30, Y = -84, Z = 18).

**Fig 11:**
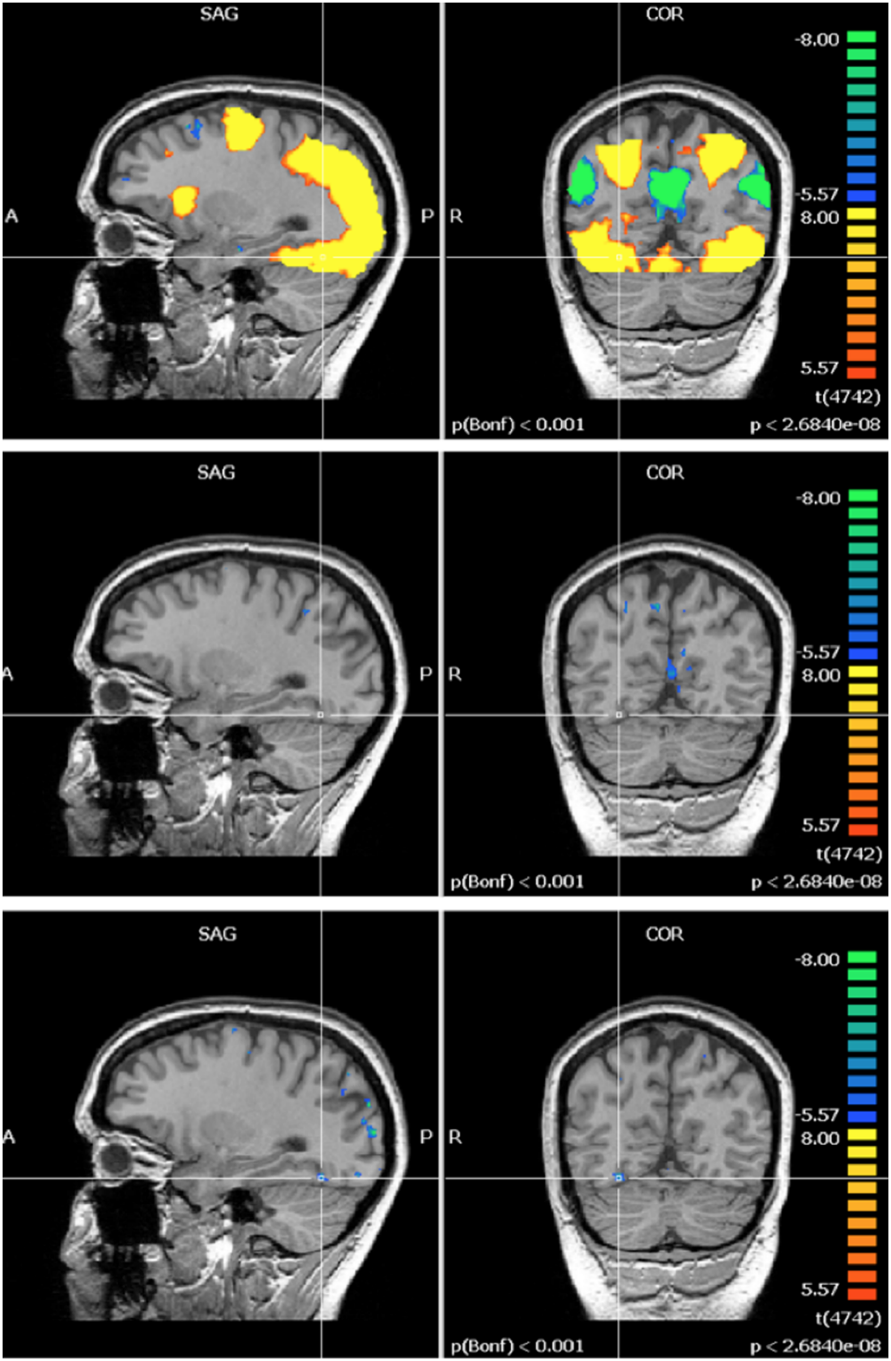
Activation in the right occipital cortex during the mental rotation task. Top) Pre-exposure (N = 21); Middle) Post- minus pre- exposure in the control group (N = 11); Bottom) Post- minus pre- exposure in the 60 Hz MF exposure group (N = 10). Results centered on the point of Talairach coordinates (X = 27, Y = -59, Z = -23).

Pre-exposure (60 Hz MF + control, N = 21) activation maps of the mental rotation task showed activation in regions associated with integration of visual and motor information, visual processing and executive function and cognitive control; which are all regions that have been linked with the mental rotation task in previous fMRI studies [37]. The multi-study GLM analysis in BrainVoyager revealed a significant post-exposure deactivation in the 60 Hz MF exposure group in the left intraparietal sulcus, and a decrease in activation in the posterior cingulate. Another multi-study GLM analysis revealed a significant increase in post-exposure activation in the control group in the occipital lobe. A repeated-measures ANOVA, conducted on the extracted beta weight values from the ROI, showed significant ‘time by exposure’ interactions. In the posterior cingulate (F = 7.629, p = 0.012), left intraparietal sulcus (F = 4.705, p = 0.043), and right occipital lobe (F = 5.742, p = 0.027), the interaction revealed a post-exposure deactivation that was significantly stronger in the 60 Hz MF exposure group compared to the control exposure group (Figs 9, 10 and 11).

**Fig 12:**
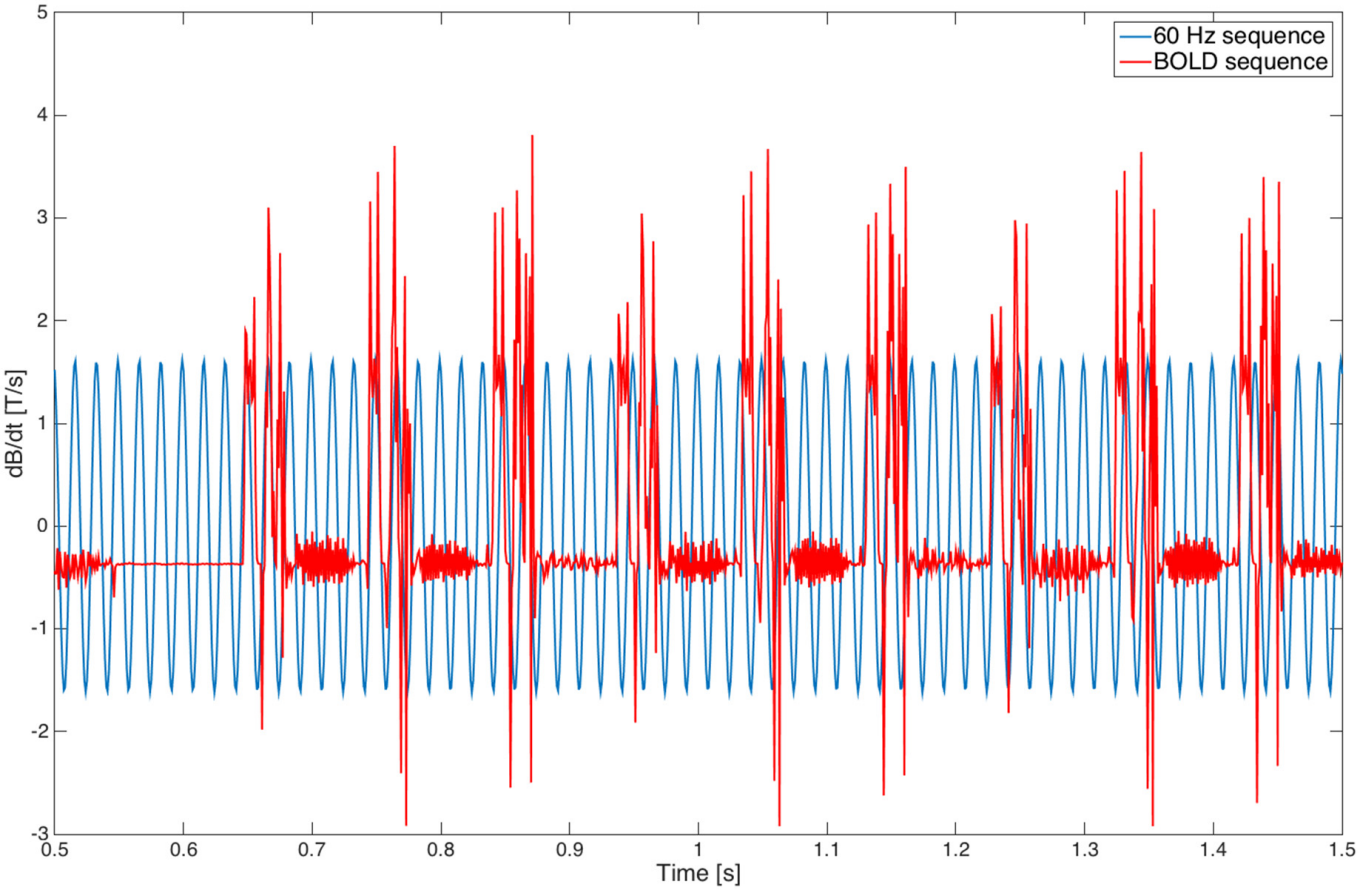
Time series of the magnetic induction measured during the 60 Hz and BOLD sequences.

**Fig 13:**
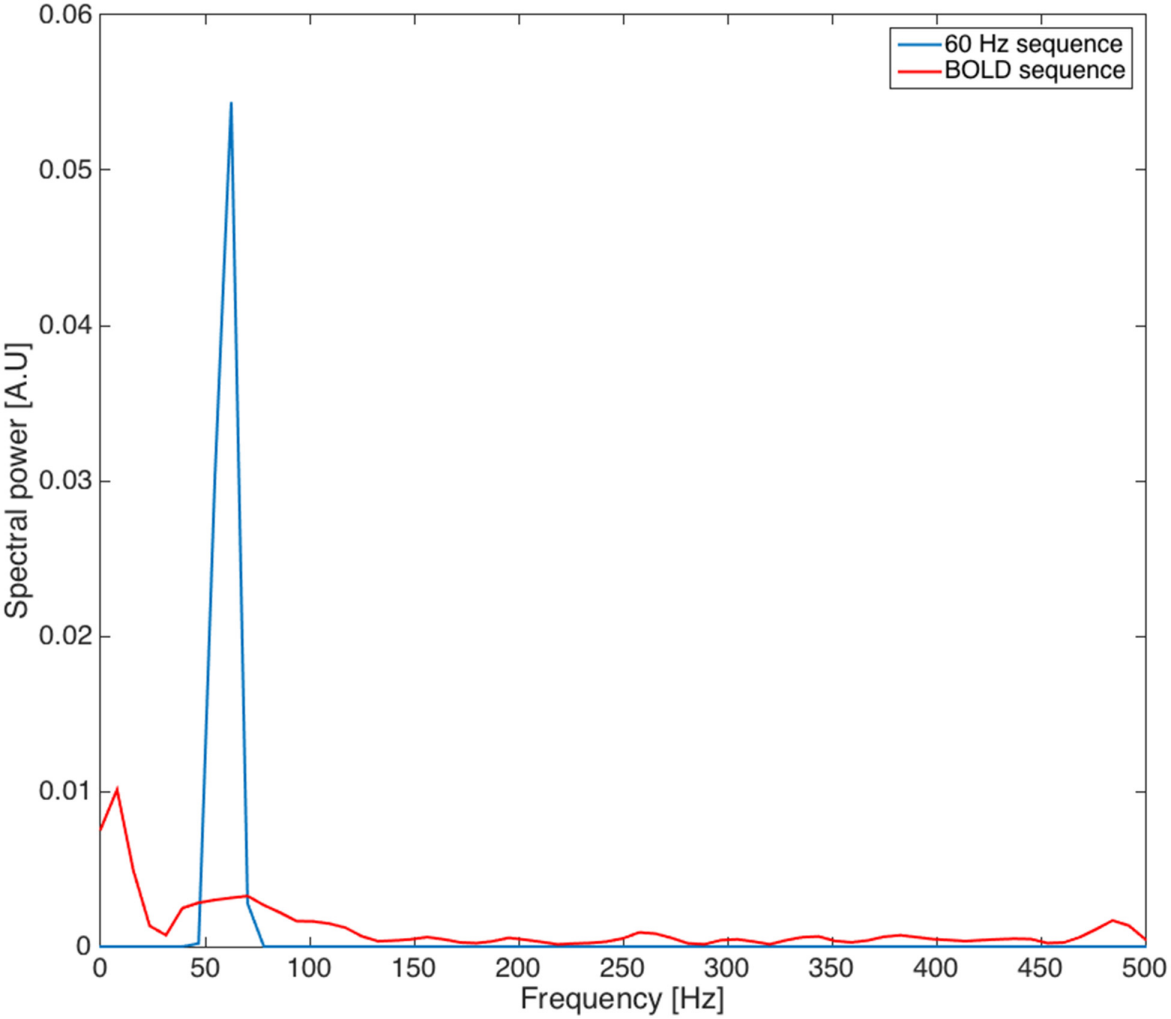
Comparison of the magnetic induction power spectrum for the 60 Hz and BOLD sequences.

In order to rule out the possible role of the gradient fields generated during the fMRI BOLD sequence, we have measured the electric field (since this is the electric field, and not the MF itself, that interacts with neuron membranes) induced by the 60 Hz MF exposure and during an fMRI BOLD sequence. We have used a custom-made MRI-compatible magnetic induction probe, placed into the MR bore at z = 13 cm to mimic the situation where subjects were exposed, using a firm foam support. Examples of corresponding time series are presented in Fig 12. The corresponding power spectrum of magnetic field induction during 60 Hz and BOLD sequences is presented in Fig 13. This probe was also used to confirm that the measured MF was corresponding to the programmed pattern delivered through the Z gradient coil of the MRI system. In both experiments, at the time of the exposure, the patient table was offset 13 cm cranially from the isocentre so that the field at the cortical level was set to be 1.8 mT in the pilot and 3 mT in the full experiment. The measured peak rate of change of the applied 60 Hz MF at this offset point were 0.959 T/s (i.e. 0.678 T/s rms) and 1.599 T/s (i.e. 1.13 T/s rms) respectively (see Fig 12 for a recording at 3 mT). Since dB/dt = 2.π.f.B (f being the frequency), at 60 Hz, these values actually correspond, as expected, to 1.8 and 3 mT respectively. Note that this induction probe was calibrated using a Single-Axis high-accuracy Hall Magnetic Field Transducer (Senis AG 0YA02F, Zurich, Switzerland).

Comparison of power spectra shows that the electric field induced by the fMRI BOLD sequence, despite a much weaker peak at 60 Hz than the 60 Hz sequence itself, has spectral components at very different frequencies. Notably, the electric field induced by the BOLD sequence has one slower component (approx. 10 Hz) on the one hand; and one at significantly higher frequencies (> 200 Hz) on the other hand. The depolarization of neuron membranes by electric fields is proportional to 1/√(1+ω^2^τ^2^) [49,50], where ω = 0032π*f* (*f* being the electric field frequency) and τ is the neuron membrane time constant, typically on the order of 10 ms. Therefore, the membrane polarization induced by the MF exposure decays with frequency, and it is unlikely that high-frequency components of the electric field induced by the fMRI BOLD sequence can explain our results. Furthermore, since both groups followed exactly the same protocols with the exception of the 60 Hz MF sequence, an effect induced by the fMRI BOLD sequence would have not shown in our results with the image subtraction performed. For the same reason, even if the 10 Hz component of the electric field induced by the fMRI BOLD sequence (see Fig 13) could possibly have a modulatory effect on functional brain activation (since this frequency component is low), effects would have not shown since the only difference between the groups was the presence or not of the 60 Hz MF exposure. Another argument supporting the idea that the gradient field are unlikely to be responsible for the observed results is the fact that magnetophosphenes (perception of flickering lights in the visual field during exposure to ELF MF), which represent the most robust effect of ELF MF exposure in the low milliTesla range, are preferably occurring at 20 Hz, with a frequency-dependant threshold (i.e. perception threshold increasing with frequency) [51,52].

## Discussion

As expected, brain regions previously reported to be activated during both finger tapping and mental rotation tasks were activated in a robust manner. These regions are thought to be involved in the coordination of motor processing, communicating and planning in the case of the finger tapping task [30]; and in visual attention, perceptual-motor coordination and visual working memory for the mental rotation task [37]. The comparison of Pre- and Post-Exposure functional images for both the finger tapping and mental rotation tasks supports the hypothesis that a 60 minute exposure to a 60 Hz, 3000 μT MF has the capability to significantly modulate functional brain activation induced by a motor or a cognitive task in narrow brain regions. Indeed, only brain regions involved in performing the involved task were modulated by the 1-hour 60 Hz MF exposure as compared to the condition where the MF was not present during the same period of time. However, the 60 Hz MF exposure did not have an effect on variability of the button press data, frequency of timing, or the performance of the mental rotation task.

At first the results seem to point at a discrepancy between the pilot and the full study, showing a deactivation following the sham treatment in the pilot work (not observed in the full study), and an increased activation following the MF treatment in the full study (not observed in the pilot). These differences might arise from the difference between the two protocols such as the different duration of the rest period between the pre- and post-exposure conditions and the MF flux density in the full study compared to the pilot study (1 hour, 3 mT; vs. 30 minutes, 1.8 mT respectively). However, it is interesting to highlight that the differences in brain activation in the exposed and sham groups were qualitatively similar in both the pilot and the full study: when compared to its pre-exposure condition, brain post-exposure activation was higher in the exposed group as compared to the sham group in both studies. Indeed, in the sham condition of the pilot study, the post-exposure level of activation was lower as compared to the pre-exposure condition, hence illustrating a reduction of the activation as a consequence of a resting period without any time-varying MF exposure. However, this relative reduction of activation between pre- and post-exposure conditions was canceled by the presence of the actual 60 Hz MF exposure. This illustrates a relative increase of post-exposure activation in the exposed group as compared to the sham exposed group.

Regarding the finger tapping task results, it is possible that the post-exposure increased brain activation in S1 and cerebellum, which reveals a result contrary to our hypothesis of decreased activation, might be related to the ‘practice effect’. Indeed, it has been shown that a finger-tapping task lasting between 5 and 10 minutes may be sufficient to induce plastic changes at the cortical level [53,54]. Therefore, we can hypothesize that this post-exposure over-activation may be caused by a modulation of synaptic plasticity, i.e., the capacity of synaptic weights to be modulated over time depending on neural network activity; and that is the neurological substrate for learning and memory [55]. In this hypothesis, 60 Hz MF exposure would interfere with changes in synaptic plasticity induced by task practice, which would result in a higher activation needed to produce the same task. It is important to mention that our protocol was not designed to investigate the hypothesis that 60 Hz MF exposure might induce changes in synaptic plasticity, therefore this remains speculative at that stage. However, there is a significant portion of the literature in neuromodulation research that has demonstrated changes in synaptic plasticity can result from the application of different neuromodulation modalities, e.g. transcranial direct/alternating current stimulation (tDCS/tACS) [56], transcranial magnetic stimulation (TMS) [57], deep brain stimulation (DBS) [58]. In addition, there are theoretical indication of the theoretical validity of potential changes in synaptic plasticity as a consequence of 60 MF exposure [59]. Therefore, the possibility that the electric currents induced in the brain by 60 Hz MF exposure is grounded in the neuroscience literature, and would be worth exploring further. More specifically, since the MF effects are expressed to a greater degree in the sensory pathways rather than in the motor pathways, it might involve that the MF exposure is modulating sensorimotor pathways. S1, a structure posterior to the central gyrus and connected to the motor cortex, is indeed directly related to the initial processing of cutaneous somatosensory information. Particularly, haptic stimulation (i.e. stimulation of the tactile sense) of the right index finger has been shown to be associated with an increase of blood flow in the contralateral S1 [60,61]. Sensory feedback is used by the cerebellum to monitor and optimize movement, which can be usually seen as increased activation in the brain region ipsilateral to the moving hand. It is also known that more widespread activation of the cerebellum is associated with higher levels of attention dedicated to the motor task [62]. Based on this knowledge, one may hypothesize that the MF exposure investigated in this study might interact with neural structures involved in simple repetitive motor tasks.

The Post- minus Pre-MF exposure results from the mental rotation task point at decreased activation in specific brain regions associated with the execution of this task (as in the finger tapping task), specifically the left intraparietal sulcus and the posterior cingulate. The main functions of the intraparietal sulcus are perceptual-motor coordination and visual attention, and is potentially involved in visuospatial working memory [63]; while the posterior cingulate deactivates during goal-directed tasks [64–67]. Decreased activation upon MF exposure was also observed in the right occipital region, specifically associated with visual processing. This decrease in functional activation could be a compensatory mechanism, that is, a rearrangement in neuronal circuits that would result in the maintenance of the same physiological outcome (e.g., task performance) in the presence of the external stimulus (60 Hz MF). Mental rotation task performance results suggest that the ELF MF exposure studied here (60 Hz, 3000 μT) modulates associated neuroprocessing, although the behavioural outcome is not altered. An interesting point to consider is that there are reported gender differences in brain activation associated with the mental rotation task [67,68]. When performance level was controlled for, females showed strong bilateral activation in the superior parietal lobule, including the intraparietal sulcus; males showed activation in the right parieto-occipital sulcus, the left intraparietal sulcus, and the left superior parietal lobe [68]. In the present study, both genders were used as subjects; however, there were an equal number of males and females in both the control (five males, five females) and 60 Hz MF exposed (five females, five males) groups, which may preclude a gender bias.

From the results obtained in this study, we can propose, as a potential mechanism of action, that MF exposure might interfere with brain synaptic plasticity [69]. There is a convergence of experimental [70] and theoretical [71,72] evidence that low-amplitude electric fields, such as those generated by ELF MFs, can impact the timing of action potentials (advance/delay). Spike timing is related to sensory stimuli encoding, as well as the reinforcement or weakening of synapses, and is for example central to the theory of spike-timing dependent plasticity (STDP), one of the most important forms of synaptic plasticity [69] and extensively characterized experimentally and theoretically. The potential impact on spike timing by ELF MF exposure is an additional argument toward a possible gradual modulation of synaptic efficacy (i.e., cumulative effect) [73]. Changes in synaptic efficacy could in turn alter local circuits’ dynamics, specifically electrical oscillations and synchronization, and might explain how 60 Hz MF exposure might ultimately result in altered network activity measured using fMRI.

Overall, these results show that functional brain activation induced by finger tapping, measured using the BOLD paradigm, is higher in the contralateral S1 and in the ipsilateral cerebellum (anterior lobe) after MF exposure as compared to after control exposure. This is consistent with the results of our pilot study in which the exposed participants had a significantly higher functional activation post- than pre-exposure in the exposed group as compared to the control group. In the case of the mental rotation task, brain functional activation is decreased post-exposure in the exposed group, compared to the control group, in the left intraparietal sulcus the posterior cingulate, and right occipital regions. It is difficult to evaluate from these results the cognitive impact of occupational exposure to 60 Hz MF, even if our results show unaltered cognitive performance despite changes in brain functional activation. This constitutes nevertheless an interesting direction of research.

It has already been demonstrated that fMRI can be used to measure ELF MF effects on neuroprocessing [48] (i.e., information processing by neural networks within the brain). However, since an MRI scanner uses different types of electromagnetic fields to produce functional images (static, kHz-time-varying MF, and radio-frequency electromagnetic fields), there is the possibility that these fields may induce confounds in this type of work, especially since the amplitude of the imaging MFs can be significantly higher than the ELF MFs under investigation. Nevertheless, if the exact same imaging sequences are used for both groups (“control” and “60 Hz MF”), they will be exposed to the exact same normal MRI and fMRI imaging magnetic fields. Consequently, if fMRI reveals any group differences, they should be attributed to the 60 Hz MF exposure or, alternatively, to a synergetic effect between the MRI imaging fields and the 60 Hz MF exposure, which would still be an effect attributed to the 60 Hz MF. Therefore, despite the use of electromagnetic fields that can be of a similar peak magnitude (but not frequency) as the ELF MFs of interest, fMRI should be a valuable imaging modality to detect potential brain functional activity modulation due to the 60 Hz MF exposure.

## Conclusion

Using fMRI to quantify brain functional activation during two different tasks (finger tapping and mental rotation) before and after exposure to a 60 Hz, 3000 μT MF, we have shown significant differences in task-dependant brain areas. It should be noted that, despite the modulation in neuroprocessing presented in this paper, the 60 Hz MF exposure did not impact speed or accuracy of the tasks, and therefore did not have any physical behavioural impact. Interestingly, fMRI was able to measure objectively the interaction of a 60 Hz MF at 3000 μT with the human brain in different motor and cognitive tasks, illustrating that fMRI is an appropriate tool to image the effects of ELF MF on human neurophysiology. Indeed, our fMRI BOLD results suggest the existence of an objectively measurable interaction between a 60 Hz, 3000 μT MF and brain activation.

Furthermore, the 60 Hz ELF MF exposure had a selective effect on brain regions associated with the tasks studied, i.e. the sensorimotor cortex in the finger tapping task; and visual attention and processing areas for the mental rotation task. Such selectivity of the exposure on specific brain areas remains to be explored. Despite this limitation, our results confirm the findings from our pilot study, providing a valuable direction for future research. Finally, the results reported are obtained after MF exposure, suggesting the existence of biological effects outlasting the duration of the exposure. The possibility that ELF MF exposure could modulate to some extent synaptic plasticity processes, resulting in lasting changes in brain activity, could be the focus of future research to shed light on the involved interaction mechanisms.
